# Bioengineering of Anti‐Inflammatory Natural Products

**DOI:** 10.1002/cmdc.202000771

**Published:** 2020-12-16

**Authors:** Lea Winand, Angela Sester, Markus Nett

**Affiliations:** ^1^ Department of Biochemical and Chemical Engineering Laboratory of Technical Biology TU Dortmund University Emil-Figge-Strasse 66 44227 Dortmund Germany; ^2^ Current address: Chair of Technical Biochemistry Technical University of Dresden Bergstrasse 66 01069 Dresden Germany

**Keywords:** anti-inflammatory, biocatalysis, biosynthesis, mutasynthesis, natural products

## Abstract

Inflammatory processes occur as a generic response of the immune system and can be triggered by various factors, such as infection with pathogenic microorganisms or damaged tissue. Due to the complexity of the inflammation process and its role in common diseases like asthma, cancer, skin disorders or Alzheimer's disease, anti‐inflammatory drugs are of high pharmaceutical interest. Nature is a rich source for compounds with anti‐inflammatory properties. Several studies have focused on the structural optimization of natural products to improve their pharmacological properties. As derivatization through total synthesis is often laborious with low yields and limited stereoselectivity, the use of biosynthetic, enzyme‐driven reactions is an attractive alternative for synthesizing and modifying complex bioactive molecules. In this minireview, we present an outline of the biotechnological methods used to derivatize anti‐inflammatory natural products, including precursor‐directed biosynthesis, mutasynthesis, combinatorial biosynthesis, as well as whole‐cell and *in vitro* biotransformation.

## Graphical abstract

**Nature's wealth of bioactive molecules**. This minireview gives an overview of biotechnological methods that can be used for the derivatization of natural anti‐inflammatory agents. It covers established techniques such as whole‐cell biotransformation and precursor‐directed biosynthesis, as well as recently developed concepts that involve enzyme engineering. Selected examples illustrate the diversity of structural modifications that are now possible through bioengineering.

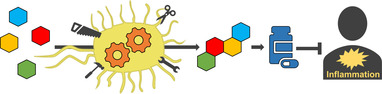

## 1 Anti‐Inflammatory Drugs from Nature

Inflammation is initiated by a multitude of external and internal triggers and it can lead to acute or chronic diseases with characteristic symptoms including pain, fever, reddening, swelling and impaired functionality.^[1]^ Some of the oldest reports go back to the Neanderthals, whose skeletons were found to carry signs of arthritis and inflammatory processes. Equally old is the human drive to discover remedies for such conditions and already the *Materia medica* from the first century AD includes respective recipes, for example, for decoctions of white willow leaves and cortex. It was though not until the end of the 18th century that scientists isolated and characterized the active compounds therein. The initially isolated salicin was described as the sugar conjugate of salicylic acid, and the latter soon found its way into treatment of fever and inflammatory rheumatoid arthritis.^[2]^ Optimization of the natural product resulted in the improved structure‐activity profile found in its derivative acetylsalicylic acid that henceforth became the drug of choice. Development of its total synthesis facilitated distribution and fostered its global success that continues until today.^[3]^ Salicylic acid is likely the most prominent example of a natural product providing the scaffold for a medicine that is frequently used in current days.

Inflammatory processes include complex physiological pathways, that also interconnect with many other signaling cascades, affecting cancer development, blood coagulation as well as immune and allergic reactions.^[4]^ There are a range of essential pathways that dictate the cascade starting from an inflammatory trigger to a complex reaction that involves unspecific and specific immune response, composed of cellular components and multiple small molecule mediators. The so‐called nonsteroidal anti‐inflammatory drugs (NSAIDs) target the cyclooxygenases (COX‐1/2) which, together with the lipoxygenases (LOX‐5/12/15), catalyze the first step from arachidonic acid towards a multitude of pro‐ but also anti‐inflammatory prostaglandins, leukotrienes and thromboxanes.^[5]^ Other major pathways involve Toll‐like receptor (TLR)‐induced and mitogen‐activated protein (MAP) kinase cascades or glucocorticoid receptors that control transcription factors, such as NFAT, NFκB or STAT3. The latter generally regulate and induce production of proinflammatory agents, including diverse interleukins (IL‐1β, IL‐6, IL‐8), TNF‐α, iNOS or COX‐2.^[6]^

Natural products played essential roles in the identification of inflammatory pathways. An illustrative example is given by the bacterial macrolides sirolimus (rapamycin) and tacrolimus (FK506). Following the discovery that both natural products bind the FK‐binding protein 12 (FKBP‐12), the respective downstream mechanism through the phosphatase calcineurin and the kinase target of rapamycin (TOR) could be clarified.^[7]^ Together with the peptide cyclosporin A, these compounds soon became essential probes to study signal transduction.^[8]^ Similarly, the plant polyphenol nordihydroguaiaretic acid and the terpenoid 3‐acetyl‐11‐keto‐β‐boswellic acid from frankincense were recently characterized as active site and allosteric inhibitors of 5‐LOX, respectively, thereby giving mechanistic insights into a known activity and revealing new potential targets for further directed drug design.^[9]^ In the light of these examples it is not surprising that about a quarter of all FDA‐approved anti‐inflammatory compounds are natural product derivatives.^[10]^

## 2 Biotechnological Methods for Natural Product Derivatization

**Figure 1:**
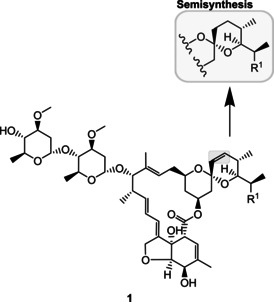
Chemical structure of avermectin B1 (R^1^=C_2_H_5_, CH_3_). Selective hydrogenation gives rise to the semisynthetic derivative ivermectin.^[16]^ The structural modification is highlighted.

Natural products are often regarded as evolutionarily optimized ligands for biological targets and receptors.[^11^, ^12^] For a therapeutic use, however, many natural products need to be improved in terms of selectivity, pharmacokinetic properties, and stability.[^12^, ^13^, ^14^] While total synthesis offers vast opportunities for chemical modifications, it can also be laborious and costly depending on the structural complexity of the target molecule. For this reason, minor variations, such as esterifications or halogenations, are in general rather introduced by chemical derivatization of a previously isolated natural product than by total synthesis.^[15]^ A noteworthy example is ivermectin, which derives from the bacterial macrolide avermectin B1 (**1**; Figure 1)^[16]^ and is used, amongst others, in the treatment of papulopustular rosacea.^[17]^

Although semisynthetic approaches are of high relevance, they are occasionally hampered by positional selectivity and the reactivity of interfering functional groups. A smart way to circumvent these issues is the use of biotechnological methods, which allow the derivatization of natural products in a regio‐ and stereocontrolled manner. The available techniques can be roughly divided into those, which make use of living cells to biosynthesize unnatural analogues and those, in which the modifications are carried out with purified enzymes under *in vitro* conditions.

### 2.1 *In vivo* methods

**Figure 2:**
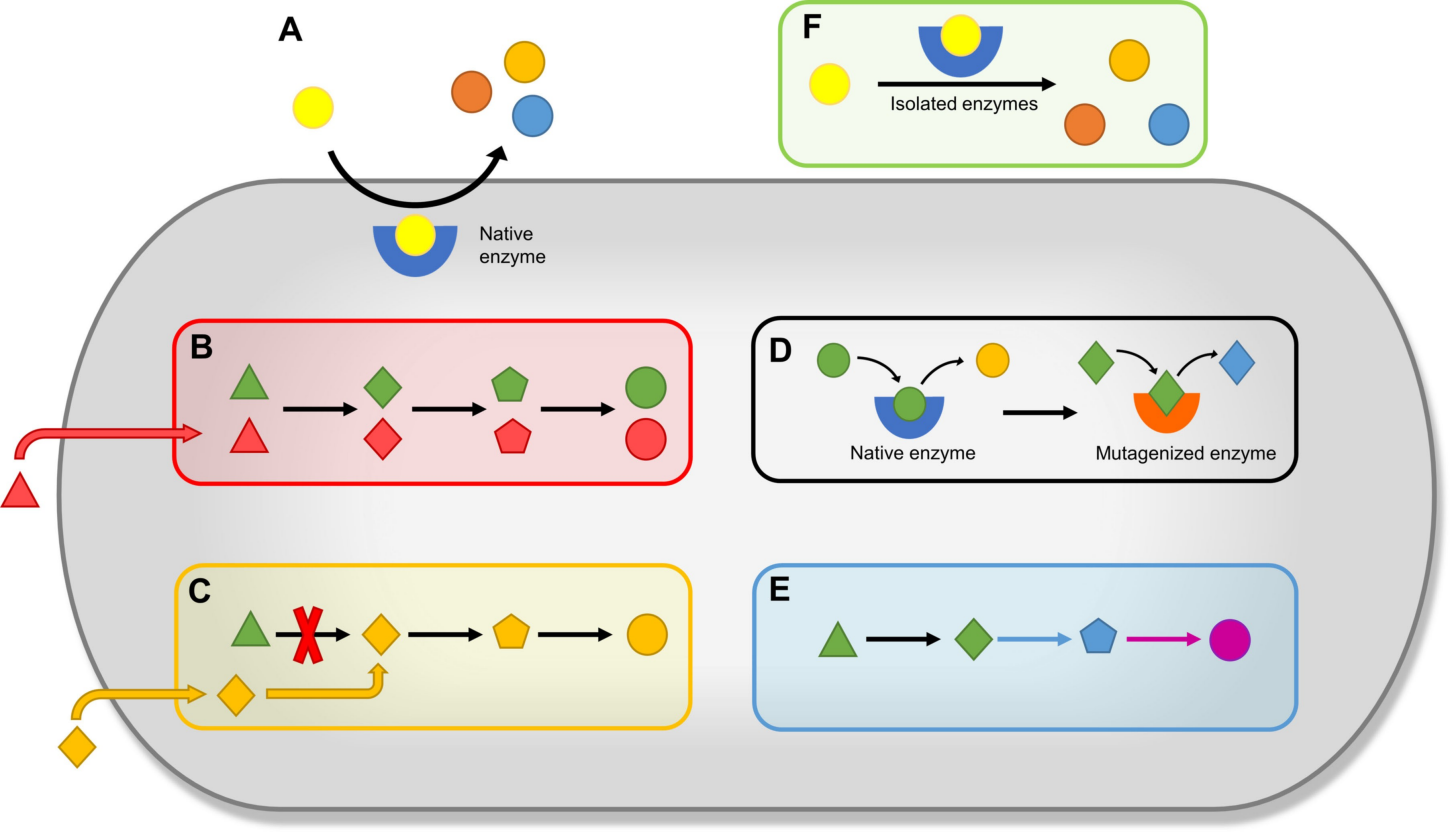
Schematic representation of biotechnological methods for natural product derivatization: A) whole‐cell biotransformation, B) precursor‐directed biosynthesis, C) mutasynthesis, D) mutaXchange, E) combinatorial biosynthesis, and F) *in vitro* biotransformation.

A well‐known method to increase the structural diversity from an isolated natural product is whole‐cell biotransformation. The molecule of interest is fed to an organism other than the producer (in most cases a fungus or a bacterium), which then carries out various site‐specific and stereospecific reactions (Figure 2A).[^18^, ^19^] Although the outcome of such a derivatization is often not foreseeable, the knowledge about microbial strains carrying out desirable transformations has accumulated over the years. Quite often, proven “biotransformers” are easily available from microbial culture collections.^[20]^ Whole‐cell biotransformation has been especially useful in the derivatization of steroid natural products and many steroid drugs as well as their precursors (e. g., hydrocortisone, 11α‐hydroxyprogesterone, 4‐androstene‐3,17‐dione) are actually manufactured in this way.^[21]^

Another easy way to obtain natural product derivatives is to supply the producer organism with analogues of biosynthetic building blocks, such as halogenated amino acids or aryl carboxylic acids. Depending on cellular uptake and the substrate tolerance of the biosynthesis enzymes, these analogues can compete with and replace the metabolically derived precursors (Figure 2B).^[22]^ This approach is known as precursor‐directed biosynthesis (PDB) and has a long tradition. For instance, it led to the discovery of the first orally active β‐lactam antibiotic.^[23]^ The main advantages of PDB lie in its broad applicability and uncomplicated implementation. On the other hand, PDB typically generates a mixture of the unmodified natural product along with the desired derivative(s), which affects the yield and results in extensive purification efforts.[^22^, ^23^]

The aforementioned issues can be circumvented with a technique called mutasynthesis. This technique presupposes the elimination of a biosynthetic precursor or intermediate by targeted gene inactivation. The resulting mutant is no longer capable to make the corresponding natural product unless the missing building block is added to complement the biosynthesis. If the mutant is instead exposed to a non‐natural surrogate of the precursor, this so‐called “mutasynthon” will be recruited for the biosynthesis and form a natural product analogue without an accompanying parental molecule (Figure 2C).[^14^, ^15^, ^23^] Due to the absence of a competing substrate, mutasynthesis generally leads to higher incorporation rates of the fed precursors when compared to PDB. However, there is also a specific limitation. Common biosynthetic building blocks, such as malonyl‐CoA or proteinogenic amino acids, are not accessible via classical mutasynthesis, because the disruption of their pathways would have lethal consequences for the cell.

Both PDB and mutasynthesis exploit the inherent promiscuity of biosynthetic enzymes. In general, the substrate flexibility of these catalysts confines the range of possible derivatizations, but this boundary can be overcome through genetic engineering. An increasing number of studies make use of biosynthesis enzymes that were mutagenized in order to expand or to alter their substrate specificity (Figure 2D).[^24^, ^25^, ^26^, ^27^]

We refer to the corresponding studies as mutaXchange, even though the term enzyme‐directed mutasynthesis is also used in the literature.^[24]^ It is necessary to differentiate between mutaXchange and the concept of combinatorial biosynthesis. MutaXchange involves the engineering of a defined natural product pathway, whereas combinatorial biosynthesis aims to mix enzymes and domains from different endogenous or exogenous pathways for the creation of new compounds (Figure 2E). The modification of functional groups, for example, the attachment of sugar or methyl moieties, is easily feasible by combinatorial biosynthesis through the overexpression of suited tailoring enzymes. In contrast, sophisticated modifications, including the recombination of core enzymes in polyketide and nonribosomal peptide biosynthesis, have long been out of reach. This situation, however, is about to change.^[28]^ The increasing availability of structural data for many biosynthetic enzymes and the ensuing mechanistic insights have recently led to the development of rational engineering strategies for the custom design of multidomain megasynth(et)ases.[^29^, ^30^]

### 2.2 *In vitro* methods

The structural diversification of natural products can also be carried out with purified enzymes in a biocatalytic approach (Figure 2F). Although *in vitro* biotransformation is analogous to whole‐cell biotransformation, it offers several advantages over the latter. As applied biocatalysts are known, they can be used in a much more directed and predictable way. Transport limitations due to poor cellular uptake of the natural product substrate are avoided and the purification of the conversion products becomes easier, as no metabolic by‐products need to be separated. It should be noted that *in vitro* biocatalysis has become quite popular in pharmaceutical industry for the design of greener, sustainable manufacturing processes. This development is also not restricted to natural product‐derived drugs, as illustrated by the enzymatic synthesis of the antihyperglycemic agent sitagliptin.^[31]^

## 3 Engineering Anti‐Inflammatory Natural Products

In the following section, we shall highlight anti‐inflammatory natural products that have been bioengineered to improve their properties (Table S1). This compilation is far from comprehensive and it is also not intended to be so. The examples were chosen to illustrate the different methods introduced in Section 2 and will mainly cover studies that have been reported since 2010.

### 3.1 Tacrolimus (FK506)

Tacrolimus (**2**) is among the clinically most relevant immunosuppressant compounds.^[32]^ In addition, it is used for the treatment of inflammatory skin diseases^[33]^ and it might have therapeutic value as a neuroprotective and neuroregenerative compound.^[34]^ Tacrolimus binds FKBP‐12 and the resulting compound–protein complex deactivates calcineurin, which hinders activation of the transcription factor NFAT. This ultimately leads to a reduced production of proinflammatory IL‐2.^[7]^

Although significant achievements have been made in the total synthesis of **2**,^[35]^ its derivatization is still a formidable task considering the structural and stereochemical complexity of this molecule. Biosynthetic approaches offer an attractive alternative and will likely become even more relevant with the increasing availability of genetically engineered strains with high tacrolimus production titers.[^36^, ^37^, ^38^, ^39^, ^40^, ^41^, ^42^, ^43^]

The bacterial natural product is assembled by a polyketide synthase (PKS)‐nonribosomal peptide synthetase (NRPS) enzyme complex.[^44^, ^45^] Its biosynthesis involves several unusual building blocks, including the shikimate‐derived metabolite (4*R*,5*R*)‐4,5‐dihydroxycyclohex‐1‐enecarboxylic acid (DHCHC)^[46]^ as well as the rare extender units methoxymalonyl‐ACP and allylmalonyl‐CoA.[^47^, ^49^] After 10 successive polyketide chain elongations starting from the DHCHC moiety, a lysine‐derived l‐pipecolate unit is attached by an NRPS. A subsequent cyclization generates the macrolide scaffold of **2**, which is then subjected to further oxidation and methylation reactions.[^44^, ^45^]

The molecular structure of **2** was extensively modified both chemically and biotechnologically to create analogues with improved stability, solubility and reduced toxicity. Structure‐activity relationship (SAR) studies indicated two sites on this natural product to be of primary interest for derivatizations. Thus, modifications at the cyclohexyl moiety do not affect the inhibition of calcineurin, but influence stability and solubility, whereas modifications in the allyl side chain directly correlate with the immunosuppressive bioactivity.[^49^, ^50^, ^51^]

**Figure 3:**
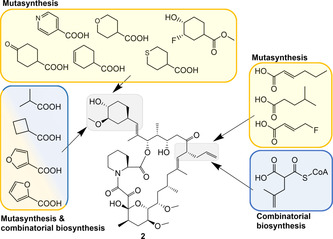
Chemical structure of tacrolimus (**2**) and some representative, unnatural precursors that were successfully incorporated by mutasynthesis and/or combinatorial biosynthesis.[^50^, ^51^, ^52^, ^53^, ^54^, ^55^, ^56^]

To alter the cyclohexyl moiety through bioengineering, the DHCHC pathway needs to be inactivated in the tacrolimus‐producing bacterium. Feeding of the mutant with cyclic carboxylic acids fuels the biosynthesis and leads to the mutasynthetic production of various derivatives (Figure 3).[^50^, ^52^, ^53^] A complementary approach is based upon the replacement of the PKS domains that are responsible for the loading of the DHCHC precursor. This domain swapping strategy was shown to increase the structural diversity that can be generated by mutasynthesis.^[50]^

The allyl side chain of **2** is biosynthetically accessible via the enzyme TcsB, an unusual β‐ketoacyl synthase. Thus, after disruption of the *tcsB* gene, unnatural extender units similar to allylmalonyl‐CoA can be channeled into tacrolimus biosynthesis. One noteworthy compound that was produced in this way is 36‐methyl‐tacrolimus.^[54]^ Although 36‐methyl‐tacrolimus did not exhibit improved immunosuppressive activities, it enhanced the neurite outgrowth, which is desirable for nerve regeneration.^[51]^ An improved production process for 36‐methyl‐tacrolimus, which omits the need for external precursor supply, was subsequently achieved by heterologous reconstitution of the isobutyrylmalonyl‐CoA pathway in the *tcsB* mutant.^[55]^ More recently, the pathway to allylmalonyl‐CoA as well as the post‐PKS tailoring reactions in tacrolimus biosynthesis have also been targeted by gene inactivation in order to introduce specific structural modifications.^[56]^

### 3.2 Prodigiosin and prodiginines

Prodigiosin (**3**) is the prototype of a family of red‐colored pigments featuring a tripyrrole ring structure.^[57]^ These pigments, which are produced by diverse bacteria, are known in the literature as prodiginines. Over the years, the prodiginines have attracted significant attention due to their apoptotic effects on malignant tumor cells.^[58]^ In several clinical studies synthetic prodiginine derivatives, such as obatoclax, were tested against different types of cancer.[^57^, ^58^] In contrast, the anti‐inflammatory properties of **3** and its analogues are less known. Various studies showed that the prodiginines delay the progression of arthritis and improve gastric inflammations by interference with inflammatory mediators, such as the IL‐2 receptor α‐chain, NFκB, nitric oxide (NO), iNOS or JNK.[^59^, ^60^, ^61^, ^62^, ^63^] *In silico* studies further suggest that the prodiginines act as COX‐2 inhibitors.^[64]^

**Figure 4:**
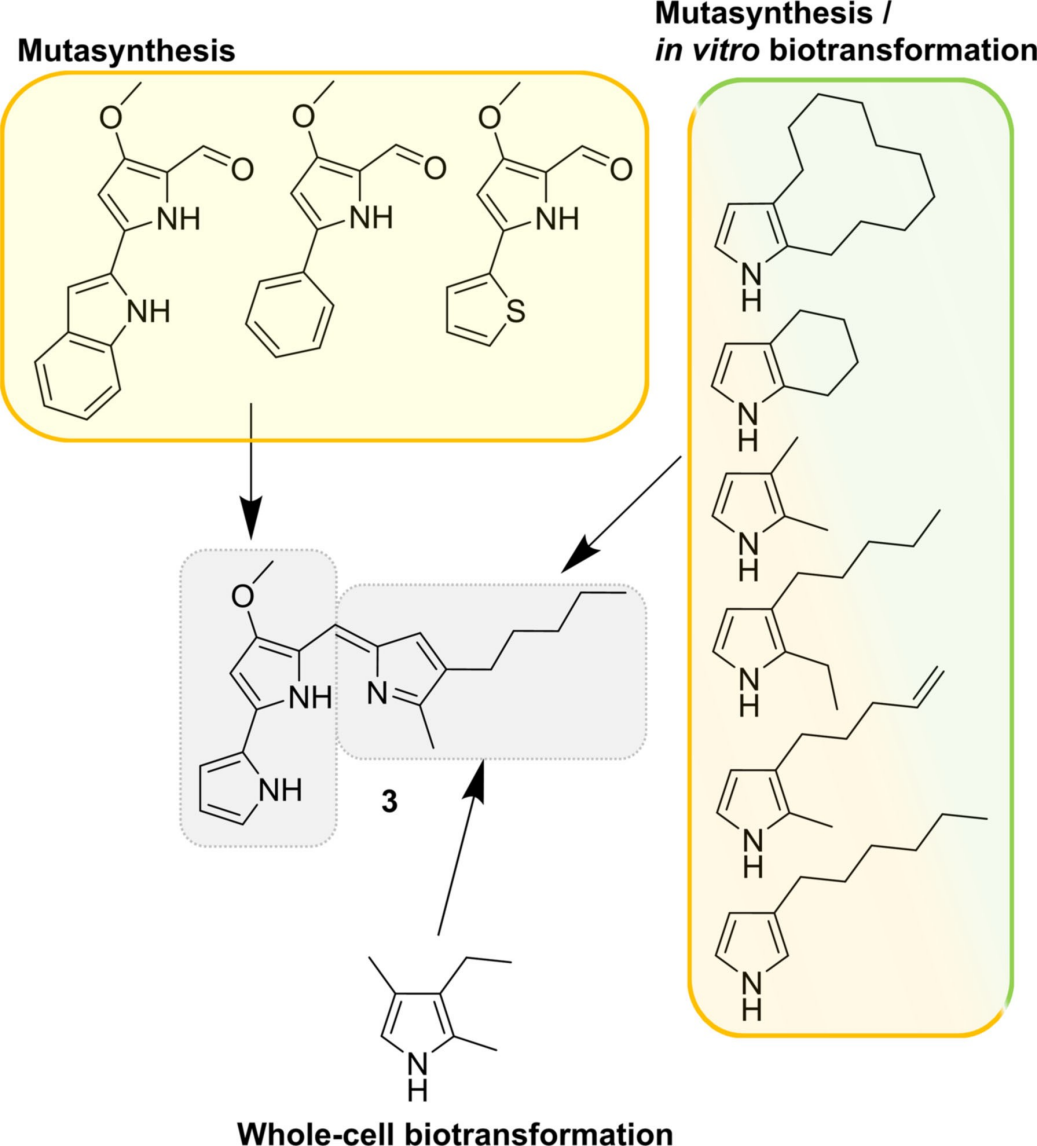
Chemical structure of prodigiosin (**3**) and some representative, unnatural precursors that were successfully incorporated by mutasynthesis, *in vitro* or whole‐cell biotransformation.[^57^, ^66^, ^67^, ^68^, ^69^, ^70^, ^71^, ^72^, ^73^, ^74^, ^75^]

Because the total syntheses of prodiginines are costly and typically involve several steps with an overall low yield,[^65^, ^66^] alternative manufacturing ways have been sought. The fermentative production of prodiginines benefits greatly from the substrate flexibility of the enzymes in the biosynthetic pathway, which enables the generation of customized analogues (Figure 4).[^66^, ^67^, ^68^, ^69^, ^70^, ^71^, ^72^, ^73^, ^74^] In brief, the characteristic tripyrrole motif of these natural products originates from the linkage of two biosynthetic intermediates, namely 4‐methoxy‐2,2′‐bipyrrole‐5‐carbaldehyde (MBC) and a monopyrrole, which varies depending on the producer organism. In prodigiosin biosynthesis, this monopyrrole is 2‐methyl‐3‐pentylpyrrole (MPP). As the enzyme PigC, which condenses the two precursors, is promiscuous, different building blocks can be incorporated contingent upon the inactivation of the MBC and MPP pathways.

The feasibility of this mutasynthetic strategy was initially demonstrated with MBC block mutants of the natural prodiginine producers *Serratia* sp.^[67]^ and *Streptomyces coelicolor*.^[68]^ Furthermore, it was shown that prodiginine derivatives can also be produced with an engineered *Escherichia coli* strain that heterologously expresses the MPP biosynthetic enzymes and the condensing enzyme PigC.^[67]^ In recent years, *Pseudomonas putida* 2440 was explored as a heterologous host for the production of prodiginine derivatives. Following the successful reconstitution of the biosynthetic pathway from *Serratia marcescens*,^[75]^ a MPP block mutant of *P. putida* was generated. Feeding of this strain with synthetic pyrroles allowed the mutasynthetic production of numerous prodigiosin derivatives.[^66^, ^71^] A novel analogue that was generated in this way showed superior anticancer activity when compared to **3** or its synthetic analogue obatoclax.^[71]^ Recently, it was demonstrated that the substrate tolerance of the flavin‐dependent dihydropyrrole oxidase PigB in the MPP pathway permits also the use of pyrrolines as alternative delivery vehicles in the mutasynthesis of **3**.^[74]^

In 2018, the production of prodiginines by whole‐cell biotransformation was reported. In this study, the key biosynthesis enzyme PigC from *S. marcescens* was overexpressed in *E*. *coli* and the recombinant strain was then used to catalyze the condensation of two synthetic substrates, MBC and 2,4‐dimethyl‐3‐ethylpyrrole (DMEP).^[72]^ The PigC‐catalyzed conversion of MBC‐ and MPP‐type substrates can also be carried out in a cell‐free, one‐pot reaction.^[66]^ Brass et al. identified an homologue of PigC in tambjamine biosynthesis. This enzyme, TamQ, has a higher substrate tolerance and activity in comparison to PigC, since it efficiently converts diverse cyclic pyrroles into prodiginines *in vitro*.^[73]^ Thus, TamQ may serve as a better biocatalyst for the production of customized prodiginines.^[73]^

### 3.3 Myxochelins

The myxochelins (**4**) are bacterial siderophores^[76]^ that show strong antiproliferative effects on leukemic cells.[^77^, ^78^] The respective activity was traced to an inhibition of the human 5‐LOX,^[78]^ which is known to act as a pro‐malignancy factor.^[79]^ Moreover, 5‐LOX is crucially involved in various inflammatory processes, making this enzyme an important drug target.^[79]^

**Figure 5:**
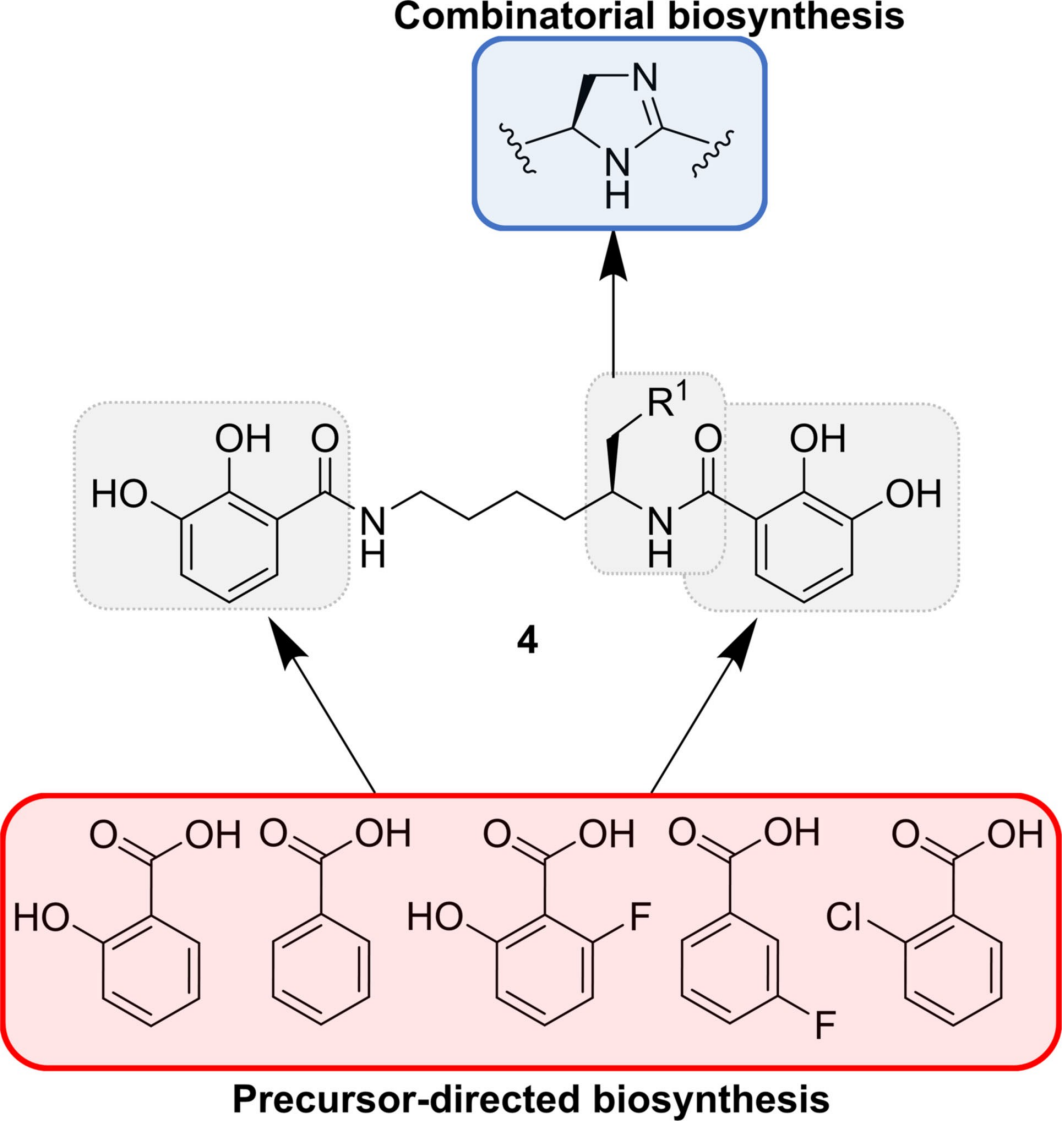
Chemical structure of myxochelin A (R^1^=OH) and B (R^1^=NH_2_) (**4**). While the two catechol moieties can be varied by precursor‐directed biosynthesis, the core region is amenable to combinatorial biosynthesis.[^85^, ^86^, ^87^]

Due to their comparatively low structural complexity, the myxochelins can be efficiently prepared by total synthesis,[^80^, ^81^, ^82^] which already enabled an extensive testing of analogues with regard to their 5‐LOX inhibitory properties.^[83]^ Noteworthy, several derivatives were also produced by biosynthetic engineering, as this approach omitted the repeated use of identical linear transformations. The corresponding studies initially focused on the two 2,3‐dihydroxybenzoate‐derived catechol units, which seemed particularly promising residues for a replacement. Consistent with the previously reported substrate flexibility of the myxochelin biosynthesis enzymes,^[84]^ various unnatural aryl carboxylic acids could be incorporated into the scaffold of **4** (Figure 5).^[85]^ Since the pathway to 2,3‐dihydroxybenzoate was not impaired, feeding of a single precursor analogue led to a randomized substitution of the two aromatic moieties. In this way, a large number of myxochelin derivatives could be prepared in a short time frame. It was further suggested that this biosynthetic peculiarity might be exploitable in a combinatorial fashion to introduce two different, unnatural building blocks at once.^[85]^ From a medicinal chemistry perspective, the generated derivatives provided important insights into the SAR of the myxochelins. It turned out that the catechol groups were dispensable for effective 5‐LOX inhibition, which was not expected in consideration of the active site iron of this enzyme.^[85]^

In a subsequent study, combinatorial biosynthesis was used to further extend the structural diversity of the myxochelins. Heterologous expression of the amidohydrolase MxcM in the myxochelin‐producing soil bacterium *Myxococcus xanthus* gave access to an imidazoline‐featuring derivative named pseudochelin A.^[86]^ Briefly, MxcM catalyzes an intramolecular condensation reaction of the β‐aminoethyl amide group in myxochelin B.^[86]^ Previous investigations showed that the lysine building block, which links the two 2,3‐dihydroxybenzoate residues, cannot be easily replaced by other amino acids.^[84]^ Late‐stage functionalization is thus likely the most promising alternative for modifications on the myxochelin core. Eventually, the two bioengineering strategies were combined to generate 5‐LOX inhibitors that are equipotent to the FDA‐approved drug zileuton.^[87]^

### 3.4 Noscapine

**Figure 6:**
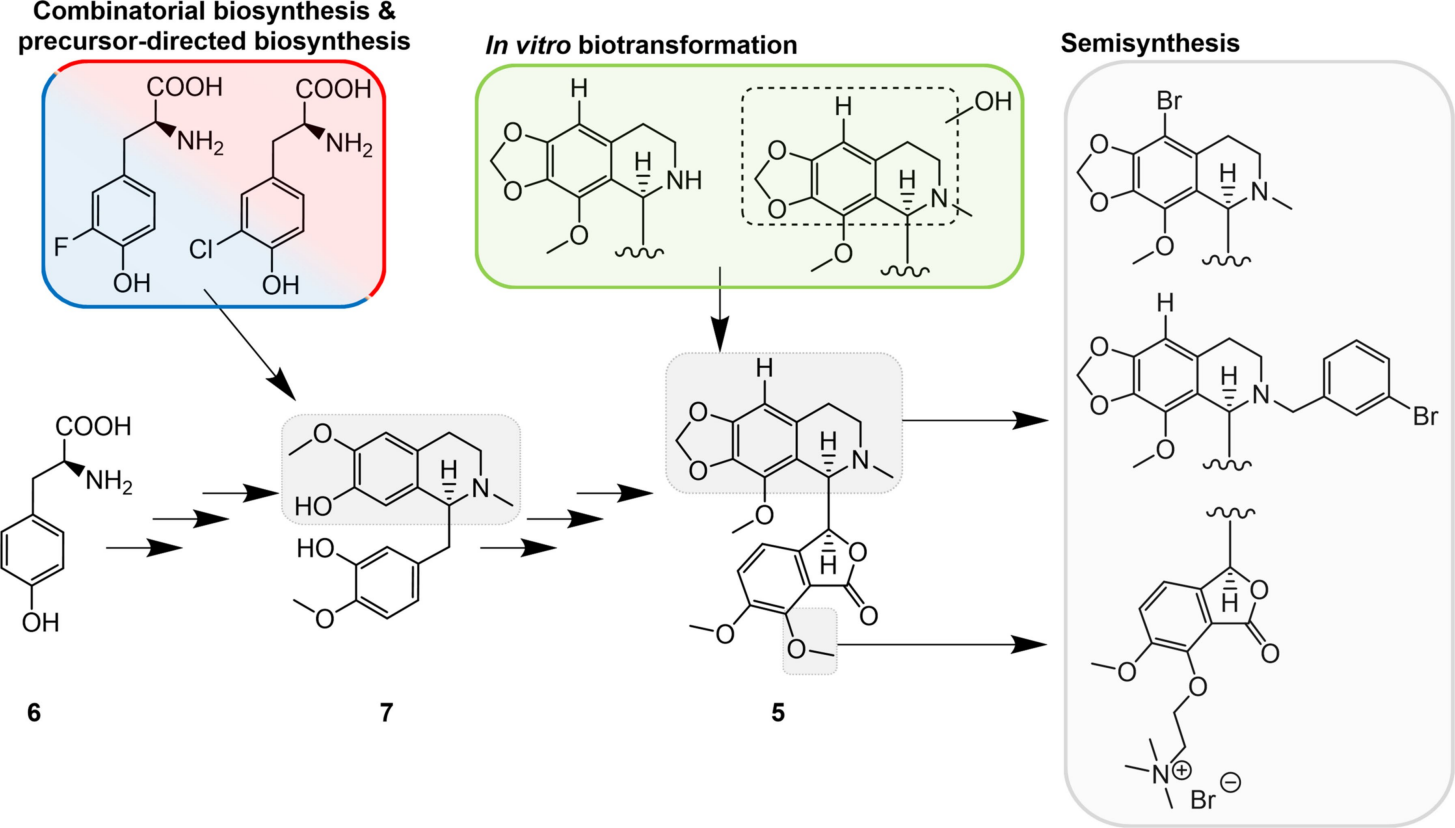
Pathway to noscapine. The biosynthesis of noscapine (**5**) starts from l‐tyrosine (**6**) and proceeds via the intermediate reticuline (**7**). Residues that were successfully modified in reticuline and noscapine by biotechnological or semisynthetic methods are highlighted.[^90^, ^91^, ^92^, ^93^, ^94^, ^95^, ^96^, ^97^, ^98^, ^99^, ^100^, ^101^, ^102^, ^105^, ^106^, ^108^]

The benzylisoquinoline alkaloid noscapine (**5**; Figure 6) was firstly characterized from opium poppy (*Papaver somniferum*) in 1817 and is, after morphine, the alkaloid with the second highest abundance in opium.[^88^, ^89^] Noscapine is mainly used as an antitussive drug because, unlike other alkaloids obtained from opium, it does not exhibit any narcotic effects. Furthermore, **5** gained increasing attention as a chemotherapeutic agent due to its potent tubulin‐binding properties and its good safety profile.^[89]^ More recently, **5** and some semisynthetic derivatives were reported to possess anti‐inflammatory activity. The tested noscapinoids were shown to inhibit the TLR‐induced release of TNF‐α, IL‐8 and NO without affecting cell viability.^[90]^ Interestingly, brominated analogues of **5** were found to be significantly more potent than the natural product, thus demonstrating the importance of derivatization.

In the past 10 years, several attempts have been made to generate noscapine analogues with favorable properties.[^91^, ^92^, ^93^, ^94^, ^95^, ^96^, ^97^, ^98^, ^99^, ^100^, ^101^, ^102^] As a consequence of the complex stereochemistry, total chemical syntheses of noscapinoids are hard to realize. Therefore, semisynthesis has become the method of choice. Several of the generated noscapine derivatives exhibit improved bioactivities or water solubility, but the nontoxic characteristic of the natural product remains.

The biotechnological production of noscapine analogues became possible following the discovery and characterization of its biosynthesis gene cluster in the opium poppy.[^103^, ^104^] The natural assembly of **5** is extremely complex. It starts from l‐tyrosine&ek, (**6**) and involves a number of biocatalytic steps via the intermediates norcoclaurine, reticuline (**7**), scoulerine, canadine and secoberbine. In 2016, the Smolke group achieved the production of **5** in a microorganism for the first time. After reconstitution of 16 biosynthetic enzymes in the yeast *Saccharomyces cerevisiae*, the alkaloid could be produced from the precursors canadine and norlaudonosoline.^[105]^ Two years later, the same group engineered an improved yeast strain that is capable of synthesizing **5** *de novo* from a simple carbon source.^[106]^ This recombinant yeast was fed with halogenated tyrosine derivatives. Although these substrates were successfully introduced into the reconstituted pathway, as evidenced by the occurrence of halogenated alkaloid intermediates, correspondingly modified noscapines could not be detected. This result could be attributed to a restricted substrate tolerance of the late pathway enzymes, low reaction efficiencies or a low substrate abundance due to the large number of catalytic steps.^[106]^

Recently, metabolic engineering of yeast allowed the production of **7**, a key intermediate in noscapine biosynthesis, with a titer of 4.6 g/L.^[107]^ The respective platform strain will certainly facilitate further endeavors for the biotechnological preparation of noscapine analogues. Another noteworthy development in this field was the use of a library of cytochrome P450 monooxygenases to selectively introduce modifications into the noscapine scaffold.^[108]^ A directed evolution of these enzymes enhanced their activity on **5**, which then enabled the production of *N*‐demethylated and different hydroxylated noscapine derivatives under *in vitro* conditions.^[108]^

### 3.5 Saponins

Among higher terrestrial plants, the saponins are widely distributed as defense compounds against herbivores and pathogens.^[110]^ Saponins are glycosides that are typically composed of a hydrophobic aglycone equipped with functional groups and hydrophilic sugar moieties, generating an amphiphilic structure with surface‐active properties.[^109^, ^110^] According to the respective aglycone, saponins are subdivided into two classes, that is, triterpenoid and steroidal saponins.^[111]^ While their surface‐active properties make saponins efficient membrane permeabilizing agents, several studies described also immunostimulatory, anticancer, antimicrobial, antiprotozoan as well as anti‐inflammatory properties.[^109^, ^110^, ^112^] It was shown that saponins affect diverse inflammatory processes. For example, some saponins are inhibitors of 5‐LOX and COX‐2, which are involved in the formation of prostaglandins and leukotrienes. Furthermore, saponins can reduce the production of TNF‐α, NFκB, STAT3, the serine/threonin‐specific protein kinase (Akt) or induce the nuclear factors NFE2L2 or Nrf2.[^110^, ^113^, ^114^, ^115^, ^116^, ^117^, ^118^, ^119^, ^120^]

**Figure 7:**
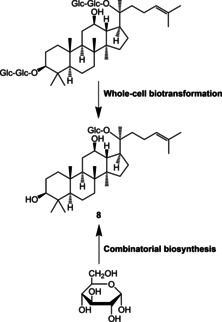
Generation of compound K (**8**) from ginsenoside Rb1 by whole‐cell biotransformation or from glucose by combinatorial biosynthesis in yeast.[^124^, ^125^, ^126^, ^127^, ^128^, ^129^, ^130^, ^131^, ^132^]

Despite these intriguing bioactivities, the therapeutic value of many naturally occurring saponins is limited due to low absorption in the human body.[^111^, ^121^, ^122^, ^123^] In contrast, rare saponins, which are less abundant in nature, are devoid of glycosyl groups and, therefore, exhibit an increased bioavailability. This fact makes biotransformations of natural saponins into rare saponins of high interest for the development of novel anti‐inflammatory drugs. One example of high relevance are saponins isolated from *Panax ginseng* named ginsenosides. Rare ginsenosides can be obtained by enzymatic deglycosylation and hydrolysis. These conversions are typically carried out with microorganisms, such as lactic acid bacteria or fungi as *Aspergillus niger*.[^124^, ^125^, ^126^, ^127^, ^128^, ^129^, ^130^] The rare ginsenoside derivative compound K (**8**) was approved by the China Food and Drug Administration for treatment and prevention of arthritis.^[124]^ Up to date, **8** is produced by deglycosylation of protopanaxadiol‐type ginsenosides (Figure 7), requiring time‐consuming cultivations of *P. ginseng* plants for substrate production.^[131]^

In 2014, a recombinant yeast strain was engineered that allowed the production of **8** from a simple carbon source.^[131]^ In the respective yeast, the native pathway for 2,3‐oxidosqualene biosynthesis, which also serves as a precursor for the biosynthesis of **8**, was extended by heterologous expression of a NADPH‐cytochrome P450 reductase from *Arabidopsis thaliana* as well as a dammarenediol‐II synthase, a cytochrome P450 and a UDP‐dependent glycosyltransferase from *P. ginseng*.^[131]^ Another combinatorial biosynthesis approach combines genes from the plants *Glycyrrhiza glabra*, *A. thaliana*, *Medicago truncatula*, *Bupleurum falcatum* and *Berberis vulgaris* in an engineered yeast, resulting in the *de novo* production of rare natural and unnatural saponins.^[132]^ The saponin production level was further increased by adding methyl‐β‐cyclodextrin to enhance the saponin export from the cells.^[132]^

## 4 Concluding Remarks

In this minireview, we have presented methods for the biotechnological derivatization of anti‐inflammatory natural products that exploit inherent or engineered enzymatic flexibility. Concepts such as mutasynthesis and combinatorial biosynthesis are not new, yet they have long been neglected in the field of medicinal chemistry. Although the specific advantages of biocatalytic modifications are clearly recognized, their use appeared restricted to niche applications, that is, specific transformations of a few, mainly microbially derived molecules. In recent years, however, biosynthetic engineering has gained momentum owing both to new structure‐driven insights into enzyme function and to the availability of convenient genome editing tools. Advances in synthetic biology have facilitated the reconstruction of complex, multigene pathways in heterologous hosts, thereby opening the door to editing the biosynthetic blueprints of plant natural products. Indeed, the future appears bright for the rational programming and customization of natural product biosynthesis, even though there are still many fundamental questions to be answered. With increasing opportunities for structural variations, biosynthetic engineering is set to become an even more valuable tool for the medicinal chemist.

## Conflict of interest

The authors declare no conflict of interest.

## Biographical Information

*Lea Winand obtained her master's degree in biochemical engineering at TU Dortmund University and currently is a PhD student in the laboratory of Prof. Nett. Her research focuses on engineering natural product pathways in myxobacteria by using a novel plasmid‐based approach. Additionally, she is developing enzymatic reaction systems for the biocatalytic production of bioactive compounds*.

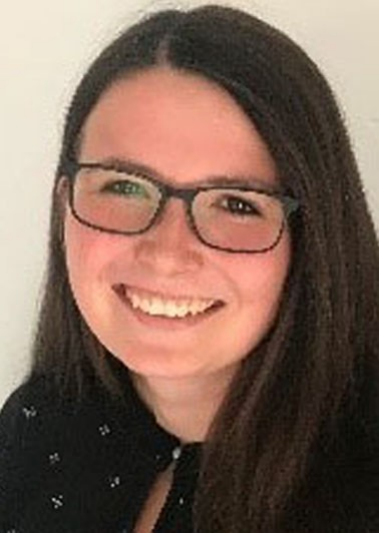

## Biographical Information

*Angela Sester is a pharmacist by training with strong interest in bioactive natural products. In her PhD projects at TU Dortmund University and the Leibniz Institute for Natural Product Research and Infection Biology, Jena, she focused on derivatization of anti‐inflammatory and antibiotic compounds through bioengineering, SAR studies and genome mining in myxobacteria*.

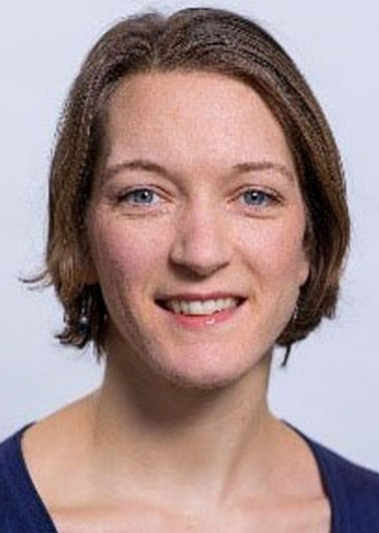

## Biographical Information

*Markus Nett is Professor for Technical Biology at TU Dortmund University. Before moving to Dortmund, he worked as a junior research group leader at the Leibniz Institute for Natural Product Research and Infection Biology in Jena. His research interests are in the field of genomics‐guided drug discovery as well as in the engineering of biosynthetic pathways from bacteria, fungi and plants*.

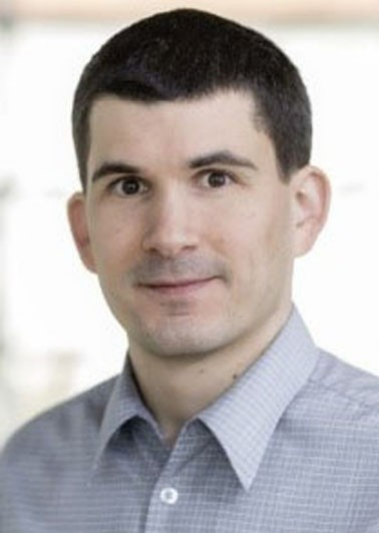

## Supporting information

As a service to our authors and readers, this journal provides supporting information supplied by the authors. Such materials are peer reviewed and may be re‐organized for online delivery, but are not copy‐edited or typeset. Technical support issues arising from supporting information (other than missing files) should be addressed to the authors.

Supplementary
