# Supplementary material for: Bioengineering of Anti‐Inflammatory Natural Products
Source: ChemMedChem. 2020 Dec 16;16(5):767–76. doi: 10.1002/cmdc.202000771 (PMC7986114; doi:10.1002/cmdc.202000771)
Supplement: Supplementary file 1 — Supplementary [file CMDC-16-767-s001.pdf]

ChemMedChem

Supporting Information

Bioengineering of Anti-Inflammatory Natural Products

Lea Winand, Angela Sester, and Markus Nett*

Supporting information

Table 1. Selected antiinflammatory natural products that were derivatized by bioengineering.

Natural product (class)	Origin	Antiinflammatory targets[1]	References	Derivatized by	References
aminocoumarin	bacterial (e.g. *Streptomyces spheroides*)	leukotriene B4, NFκB	[1, 2]	mutasynthesis	[3–5]
				combinatorial biosynthesis	[6–9]
				in vitro biotransformation	[4,10,11]
avermectin	bacterial (e.g. *Streptomyces avermitilis*)	NFκB, MAPK, IL-1b, IL-10, TNF-α, iNOS, COX-2	[12, 13]	precursor-directed biosynthesis	[14]
				mutasynthesis	[15–17]
				combinatorial biosynthesis	[18–24]
				whole cell biotransformation	[25–27]
				in vitro biotransformation	[25,26]
baicalin	plant (e.g. *Scutellaria radix*)	NO, Nrf2	[28]	whole cell biotransformation	[29]
				in vitro biotransformation	[28,30]
corticosteroid	mammal, fungal	GR, NFκB, AP-1, STAT, C/EBP, NFAT	[31, 32]	combinatorial biosynthesis	[33]
				in vitro biotransformation	[34]
				whole cell biotransformation	[35–38]
coumarin	plant (e.g. *Angelica dahurica*)	LOX, COX, iNOS	[39, 40]	combinatorial biosynthesis	[41–44]
				whole cell biotransformation	[45–48]
				in vitro biotransformation	[49,50]
curcumin	plant (e.g. *Curcuma longa)*	Nrf2, NFκB, STAT3, COX-1, 5-LOX, mPGES-1	[51–54]	precursor-directed biosynthesis	[55]
				combinatorial biosynthesis	[56]
cyclosporine	fungal (e.g. *Tolypocladium inflatum*)	IL-2, IL-12, IL-10, NO, MPO	[57–60]	precursor-directed biosynthesis	[61]
				whole cell biotransformation	[62]
erythromycin	bacterial (e.g. *Saccharopolyspora erythraea*)	IL-8	[63]	precursor-directed biosynthesis	[64–68]
				combinatorial biosynthesis	[68]
				mutaXchange	[69]
kaempferol	plant (e.g. *Ginkgo biloba*)	IL-5, IL-13, IL-6, IL-1ß, IL-18, TNF-α, NFκB, Nrf2, TLR4	[70, 71]	combinatorial biosynthesis	[72–74]
				whole cell biotransformation	[75]
manumycin	bacterial (e.g. *Streptomyces* spp.)	caspase 1, IL-1ß, IL-6, IL-8, IL-18	[76, 77]	precursor-directed biosynthesis	[78–80]
				mutasynthesis	[80]
mycophenolic acid	fungal (e.g. *Penicillium* spp.)	MPO, ADA, NOS, TNF-α, IL-1ß	[81]	*in vitro* biotransformation	[82]

Natural product (class)	Origin	Antiinflammatory targets[1]	References	Derivatized by	References
myxochelin	bacterial (e.g. *Myxococcus xanthus*)	5-LOX	[83, 84]	precursor-directed biosynthesis	[83,84]
				combinatorial biosynthesis	[83,85]
noscapine	plant (e.g. *Papaver somniferum*)	TNF-α, CXCL10, NFκB, JNK pathway, HIF-1α	[86]	precursor-directed biosynthesis	[87]
				combinatorial biosynthesis	[87,88]
				in vitro biotransformation	[89]
prodiginine	bacterial (e.g. *Serratia* spp.)	IL-2 receptor alpha chain, NFκB, iNOS, JNK, COX-2	[90–95]	mutasynthesis	[96–100]
				combinatorial biosynthesis	[101]
				in vitro biotransformation	[96,98,102,103]
				whole cell biotransformation	[104]
rapamycin	bacterial (e.g. *Streptomyces hygroscopicus*)	mTOR, TNF-α, IL-1ß	[105]	precursor-directed biosynthesis	[106–108]
				mutasynthesis	[109,110]
				whole cell biotransformation	[111]
saponin	plant (e.g. *Panax ginseng*)	Nrf2, IL-8, 5-LOX, COX-2, TNF-α, NFκB, STAT3, Akt	[112-114]	combinatorial biosynthesis	[115,116]
				whole cell biotransformation	[117–123]
tacrolimus	bacterial (e.g. *Streptomyces tsukubaensis*)	FKBP-12, calcineurin, IL-2	[124]	mutasynthesis	[125–128]
				combinatorial biosynthesis	[129]
				whole cell biotransformation	[130,131]

[1]Abbreviations: ADA = adenosine deaminase, Akt = protein kinase B, AP-1 = activator protein 1, C/EBP = CCAAT/enhancer-binding protein, COX = cyclooxygenase, CXCL10 = C-X-C motif chemokine ligand 10 (synonymous: IP-10 = interferon gamma-induced protein 10), GR = glucocorticoid receptor, HIF-1α = hypoxia-inducible factor 1-α, IL = interleukin, iNOS = inducible nitric oxide synthase, JNK = c-Jun N-terminal kinase, LOX = lipoxygenase, MAPK = mitogen-activated protein kinase, mPGES = microsomal prostaglandin E synthase, MPO = myeloperoxidase, mTOR = mammalian target of rapamycin, NFκB = nuclear factor κB, Nrf2 = Nuclear factor erythroid 2-related factor 2, NFAT = nuclear factor of activated T cells, STAT3 = Signal transducer and activator of transcription 3, TLR = Toll-like receptor, TNF-α = tumor necrosis factor α.

References

[1] E. Tsujii, N. Shigematsu, H. Hatanaka, M. Yamashita, M. Okamoto, M. Okuhara, *J. Antibiot.* **1992**, *45*, 1958–1960.
[2] T. Zheng, A. L. S. M. Noh, H. Park, M. Yim, *Biochem. Pharmacol.* **2013**, *85*, 417–425.
[3] C. Anderle, S. Hennig, B. Kammerer, S.-M. Li, L. Wessjohann, B. Gust, L. Heide, *Chem. Bio.* **2007**, *14*, 955–967.
[4] U. Galm, M. A. Dessoy, J. Schmidt, L. A. Wessjohann, L. Heide, *Chem. Biol.* **2004**, *11*, 173–183.
[5] U. Galm, S. Heller, S. Shapiro, M. Page, S.-M. Li, L. Heide, *Antimicrob. Agents Chemother.* **2004**, *48*, 1307–1312.
[6] R. H. Flatman, A. Eustaquio, S.-M. Li, L. Heide, A. Maxwell, *Antimicrob. Agents Chemother.* **2006**, *50*, 1136–1142.
[7] A. S. Eustáquio, B. Gust, S.-M. Li, S. Pelzer, W. Wohlleben, K. F. Chater, L. Heide, *Chem. Biol.* **2004**, *11*, 1561–1572.
[8] A. S. Eustáquio, B. Gust, T. Luft, S.-M. Li, K. F. Chater, L. Heide, *Chem. Biol.* **2003**, *10*, 279–288.
[9] M. Wolpert, L. Heide, B. Kammerer, B. Gust, *Chembiochem* **2008**, *9*, 603–612.
[10] C. L. Freel Meyers, M. Oberthür, L. Heide, D. Kahne, C. T. Walsh, *Biochemistry* **2004**, *43*, 15022–15036.
[11] H. Xu, L. Heide, S.-M. Li, *Chem. Biol.* **2004**, *11*, 655–662.
[12] X. Ci, H. Li, Q. Yu, X. Zhang, L. Yu, N. Chen, Y. Song, X. Deng, *Fundam. Clin. Pharmacol.* **2009**, *23*, 449–455.
[13] X. Zhang, Y. Song, X. Ci, N. An, Y. Ju, H. Li, X. Wang, C. Han, J. Cui, X. Deng, *Inflamm. res.* **2008**, *57*, 524–529.
[14] T. S. Chen, E. S. Inamine, O. D. Hensens, D. Zink, D. A. Ostlind, *Arch. Biochem. Biophys.* **1989**, *269*, 544–547.
[15] C. J. Dutton, S. P. Gibson, A. C. Goudie, K. S. Holdom, M. S. Pacey, J. C. Ruddock, J. D. Bu'Lock, M. K. Richards, *J. Antibiot.* **1991**, *44*, 357–365.
[16] E. W. Hafner, B. W. Holley, K. S. Holdom, S. E. Lee, R. G. Wax, D. Beck, H. A. McArthur, W. C. Wernau, *J. Antibiot.* **1991**, *44*, 349–356.
[17] C. D. Denoya, R. W. Fedechko, E. W. Hafner, H. A. McArthur, M. R. Morgenstern, D. D. Skinner, K. Stutzman-Engwall, R. G. Wax, W. C. Wernau, *J. Bacteriol.* **1995**, *177*, 3504–3511.
[18] T. A. Cropp, D. J. Wilson, K. A. Reynolds, *Nat. Biotechnol.* **2000**, *18*, 980–983.
[19] J.-B. Wang, H.-X. Pan, G.-L. Tang, *Bioorg. Med. Chem. Lett.* **2011**, *21*, 3320–3323.
[20] M. Li, Z. Chen, X. Lin, X. Zhang, Y. Song, Y. Wen, J. Li, *Bioorg. Med. Chem. Lett.* **2008**, *18*, 5359–5363.
[21] S. Gaisser et al., *Org. Biomol. Chem.* **2003**, *1*, 2840.
[22] P. Sun, Q. Zhao, Z. Wu, W. Zhang, W. Liu, *J. Nat. Prod.* **2015**, *78*, 301–305.
[23] J. Zhang, Y.-J. Yan, J. An, S.-X. Huang, X.-J. Wang, W.-S. Xiang, *Microb. Cell Fact.* **2015**, *14*, 1040.
[24] M.-S. Kim, W.-J. Cho, M. C. Song, S.-W. Park, K. Kim, E. Kim, N. Lee, S.-J. Nam, K.-H. Oh, Y. J. Yoon, *Microb. Cell Fact.* **2017**, *16*, 361.
[25] V. Jungmann, I. Molnár, P. E. Hammer, D. S. Hill, R. Zirkle, T. G. Buckel, D. Buckel, J. M. Ligon, J. P. Pachlatko, *Appl. Environ. Microbiol.* **2005**, *71*, 6968–6976.
[26] I. Molnár, D. S. Hill, R. Zirkle, P. E. Hammer, F. Gross, T. G. Buckel, V. Jungmann, J. P. Pachlatko, J. M. Ligon, *Appl. Environ. Microbiol.* **2005**, *71*, 6977–6985.
[27] A. Trefzer et al., *Appl. Environ. Microbiol.* **2007**, *73*, 4317–4325.
[28] K. H. Kim, Y.-D. Park, H. Park, K.-O. Moon, K.-T. Ha, N.-I. Baek, C.-S. Park, M. Joo, J. Cha, *Eur. J. Pharmacol.* **2014**, *744*, 147–156.
[29] D. H. Han, Y. Lee, J.-H. Ahn, *J. Microbiol. Biotechnol.* **2016**, *26*, 1918–1923.
[30] X. Xin, M. Zhang, X.-F. Li, G. Zhao, *J. Agr. Food Chem.* **2019**, *67*, 11684–11693.
[31] P. J. Barnes, *Br. J. Pharmacol.* **2006**, *148*, 245–254.
[32] D. W. Cain, J. A. Cidlowski, *Nat. Rev. Immunol.* **2017**, *17*, 233–247.
[33] F. M. Szczebara et al., *Nat. Biotechnol.* **2003**, *21*, 143–149.
[34] K. Suzuki, K.-i. Sanga, Y. Chikaoka, E. Itagaki, *Biochim. Biophys. Acta* **1993**, *1203*, 215–223.
[35] A. Swizdor, T. Kolek, A. Panek, N. Milecka, *Curr. Org. Chem.* **2012**, *16*, 2551–2582.
[36] J. Yang, S. Yang, Y.-L. Yang, H. Zheng, L. Weng, L. Liu, *J. Mol. Catal., B Enzym.* **2007**, *47*, 155–158.
[37] E. S. Spady, T. P. Wyche, N. J. Rollins, J. Clardy, J. C. Way, P. A. Silver, *Chembiochem* **2018**, *19*, 1827–1833.
[38] J. Wang, Y. Zhang, H. Liu, Y. Shang, L. Zhou, P. Wei, W.-B. Yin, Z. Deng, X. Qu, Q. Zhou, *Nat. Commun.* **2019**, *10*, 3378.
[39] H. S. Ban, S. S. Lim, K. Suzuki, S. H. Jung, S. Lee, Y. S. Lee, K. H. Shin, K. Ohuchi, *Planta Med.* **2003**, *69*, 408–412.
[40] K. N. Venugopala, V. Rashmi, B. Odhav, *Biomed Res. Int.* **2013**, *2013*, 963248.
[41] Y. Lin, X. Sun, Q. Yuan, Y. Yan, *Metab. Eng.* **2013**, *18*, 69–77.
[42] Y. Lin, X. Shen, Q. Yuan, Y. Yan, *Nat. Commun.* **2013**, *4*, 2603.
[43] S.-M. Yang, G. Y. Shim, B.-G. Kim, J.-H. Ahn, *Microb. Cell Fact.* **2015**, *14*, 65.
[44] L. L. Chu, R. P. Pandey, H. N. Lim, H. J. Jung, N. H. Thuan, T.-S. Kim, J. K. Sohng, *J. Biol. Eng.* **2017**, *11*, 15.
[45] S. Marumoto, M. Miyazawa, *Bioorg. Med. Chem.* **2010**, *18*, 455–459.
[46] L. Zhou, T. Tian, B. Xue, L. Song, L. Liu, R. Yu, *Biosci. Biotechnol. Biochem.* **2012**, *76*, 1008–1010.
[47] J. S. d. Nascimento, W. E. R. Núñez, V. H. P. D. Santos, J. Aleu, S. Cunha, E. d. O. Silva, *Molecules* **2019**, *24*.
[48] Ambreen, S. Haque, V. Singh, D. Katiyar, M. T. Ali Khan, V. Tripathi, H. El Enshasy, M. Pasupuleti, B. N. Mishra, *Process Biochem.* **2019**, *87*, 138–144.
[49] K. H. Kim, H. Park, H. J. Park, K.-H. Choi, R. T. Sadikot, J. Cha, M. Joo, *Sci. Rep.* **2016**, *6*, 29956.
[50] S. Park, K. Moon, C.-S. Park, D.-H. Jung, J. Cha, *J. Microbiol. Biotechnol.* **2018**, *28*, 566–570.

[51] E. Balogun, M. Hoque, P. Gong, E. Killeen, C. J. Green, R. Foresti, J. Alam, R. Motterlini, *Biochem. J.* **2003**, *371*, 887–895.
[52] A. L. Kasinski et al., *Mol. Pharmacol.* **2008**, *74*, 654–661.
[53] T. Choudhuri, S. Pal, M. L. Agwarwal, T. Das, G. Sa, *FEBS Lett.* **2002**, *512*, 334–340.
[54] A. Koeberle, H. Northoff, O. Werz, *Mol. Cancer Ther.* **2009**, *8*, 2348–2355.
[55] Y. Katsuyama, Y. Hirose, N. Funa, Y. Ohnishi, S. Horinouchi, *Biosci. Biotechnol. Biochem.* **2010**, *74*, 641–645.
[56] S. Horinouchi, *J. Antibiot.* **2008**, *61*, 709–728.
[57] E. Monguilhott Dalmarco, T. S. Fröde, Y. S. Medeiros, *Transpl. Immunol.* **2004**, *12*, 151–157.
[58] S. Steiner, C. Daniel, A. Fischer, I. Atreya, S. Hirschmann, M. Waldner, H. Neumann, M. Neurath, R. Atreya, B. Weigmann, *Arch. Immunol. Ther. Exp.* **2015**, *63*, 53–63.
[59] K. Pino-Lagos, P. Michea, D. Sauma, A. Alba, J. Morales, M. R. Bono, A. Fierro, M. Rosemblatt, *Biol. Res.* **2010**, *43*.
[60] B. Yavuz, S. Bozdağ Pehlivan, N. Unlü, *Sci. World J.* **2012**, *2012*, 194848.
[61] R. Traber, H. Hofmann, H. Kobel, *J. Antibiot.* **1989**, *42*, 591–597.
[62] M. Kuhnt, F. Bitsch, J. France, H. Hofmann, J. J. Sanglier, R. Traber, *J. Antibiot.* **1996**, *49*, 781–787.
[63] H. Takizawa et al., *Am. J. Respir. Crit. Care Med.* **1997**, *156*, 266–271.
[64] J. R. Jacobsen, C. R. Hutchinson, D. E. Cane, C. Khosla, *Science* **1997**, *277*, 367–369.
[65] D. E. Cane, F. Kudo, K. Kinoshita, C. Khosla, *Chem. Biol.* **2002**, *9*, 131–142.
[66] R. J. M. Goss, H. Hong, *Chem. comm.* **2005**, 3983–3985.
[67] C. J. B. Harvey, J. D. Puglisi, V. S. Pande, D. E. Cane, C. Khosla, *J. Am. Chem. Soc.* **2012**, *134*, 12259–12265.
[68] M. S. Pacey, J. P. Dirlam, R. W. Geldart, P. F. Leadlay, H. A. McArthur, E. L. McCormick, R. A. Monday, T. N. O'Connell, J. Staunton, T. J. Winchester, *J. Antibiot.* **1998**, *51*, 1029–1034.
[69] U. Sundermann, K. Bravo-Rodriguez, S. Klopries, S. Kushnir, H. Gomez, E. Sanchez-Garcia, F. Schulz, *ACS Chem. Biol.* **2013**, *8*, 443–450.
[70] W. Alam, H. Khan, M. A. Shah, O. Cauli, L. Saso, *Molecules* **2020**, *25*.
[71] M. Molitorisova, M. Sutovska, I. Kazimierova, J. Barborikova, M. Joskova, E. Novakova, S. Franova, *Eur. J. Pharmacol.* **2020**, 173698.
[72] X. Lyu, G. Zhao, K. R. Ng, R. Mark, W. N. Chen, *J. Agr. Food Chem.* **2019**, *67*, 5596–5606.
[73] L. Duan, W. Ding, X. Liu, X. Cheng, J. Cai, E. Hua, H. Jiang, *Microb. Cell Fact.* **2017**, *16*, 165.
[74] I. Miyahisa, N. Funa, Y. Ohnishi, S. Martens, T. Moriguchi, S. Horinouchi, *Appl. Environ. Microbiol.* **2006**, *71*, 53–58.
[75] C. Ruprecht, F. Bönisch, N. Ilmberger, T. V. Heyer, E. T. K. Haupt, W. R. Streit, U. Rabausch, *Metab. Eng.* **2019**, *55*, 212–219.
[76] T. Tanaka, E. Tsukuda, Y. Uosaki, Y. Matsuda, *J. Antibiot.* **1996**, *49*, 1085–1090.
[77] J. Hrdý, L. Súkeníková, P. Petrásková, O. Novotná, D. Kahoun, M. Petříček, A. Chroňáková, K. Petříčková, *Microorganisms* **2020**, *8*.
[78] R. Thiericke, H.-J. Langer, A. Zeeck, *J. Chem. Soc., Perkin Trans. 1* **1989**, 851.
[79] R. Thiericke, A. Zeeck, *J. Chem. Soc., Perkin Trans. 1* **1988**, 2123.
[80] S. Pospíšil, K. Petříčková, P. Sedmera, P. Halada, J. Olšovská, M. Petříček, *J. Appl. Microbiol* **2011**, *111*, 1116–1128.
[81] M. G. Beduschi, C. L. Guimarães, Z. S. Buss, E. M. Dalmarco, *Inflammation* **2013**, *36*, 729–737.
[82] W. Zhang et al., *Chembiochem* **2015**, *16*, 565–569.
[83] A. Sester, L. Winand, S. Pace, W. Hiller, O. Werz, M. Nett, *J. Nat. Prod.* **2019**, *82*, 2544–2549.
[84] J. Korp, S. König, S. Schieferdecker, H.-M. Dahse, G. M. König, O. Werz, M. Nett, *Chembiochem* **2015**, *16*, 2445–2450.
[85] J. Korp, L. Winand, A. Sester, M. Nett, *Appl. Environ. Microbiol.* **2018**, *84*.
[86] S. Zughaier, P. Karna, D. Stephens, R. Aneja, *PloS one* **2010**, *5*, e9165.
[87] Y. Li, S. Li, K. Thodey, I. Trenchard, A. Cravens, C. D. Smolke, *Proc. Natl. Acad. Sci. USA* **2018**, *115*, E3922-E3931.
[88] Y. Li, C. D. Smolke, *Nat. Commun.* **2016**, *7*, 12137.
[89] L. Richards, A. Lutz, D. K. Chalmers, A. Jarrold, T. Bowser, G. W. Stevens, S. L. Gras, *Biotechnol. Rep.* **2019**, *24*, e00372.
[90] S. B. Han, S. H. Park, Y. J. Jeon, Y. K. Kim, H. M. Kim, K. H. Yang, *J. Pharm. Exp. Ther.* **2001**, *299*, 415–425.
[91] C.-C. Chang, Y.-H. Wang, C.-M. Chern, K.-T. Liou, Y.-C. Hou, Y.-T. Peng, Y.-C. Shen, *Toxicol. Appl. Pharmacol.* **2011**, *257*, 137–147.
[92] A. Cuevas, N. Saavedra, L. A. Salazar, M. F. Cavalcante, J. C. Silva, D. S. P. Abdalla, *Int. J. Mol. Sci.* **2020**, *21*.
[93] J.-E. Huh, J.-H. Yim, H.-K. Lee, E.-Y. Moon, D.-K. Rhee, S. Pyo, *Int. Immunopharmacol.* **2007**, *7*, 1825–1833.
[94] M. S. Abdelfattah, M. I. Y. Elmallah, H. Y. Ebrahim, R. S. Almeer, R. M. A. Eltanany, A. E. Abdel Moneim, *PloS one* **2019**, *14*, e0216737.
[95] P. S. Krishna, K. Vani, M. R. Prasad, B. Samatha, N. S. V. S. S. S. L. H. Bindu, M. A. S. Charya, P. Reddy Shetty, *SpringerPlus* **2013**, *2*, 172.
[96] A. S. Klein et al., *ACS Synth. Biol.* **2017**, *6*, 1757–1765.
[97] S. R. Chawrai, N. R. Williamson, G. P. C. Salmond, F. J. Leeper, *Chem. comm.* **2008**, 1862–1864.
[98] A. S. Klein, H. U. C. Brass, D. P. Klebl, T. Classen, A. Loeschcke, T. Drepper, S. Sievers, K.-E. Jaeger, J. Pietruszka, *Chembiochem* **2018**, *19*, 1545–1552.
[99] M. Couturier, H. D. Bhalara, S. R. Chawrai, R. Monson, N. R. Williamson, G. P. C. Salmond, F. J. Leeper, *Chembiochem* **2020**, *21*, 523–530.
[100] S. W. Haynes, P. K. Sydor, A. E. Stanley, L. Song, G. L. Challis, *Chem. comm.* **2008**, 1865–1867.
[101] S. Mo, B. S. Kim, K. A. Reynolds, *Chem. Biol.* **2005**, *12*, 191–200.
[102] S. R. Chawrai, N. R. Williamson, T. Mahendiran, G. P. C. Salmond, F. J. Leeper, *Chem. Sci.* **2012**, *3*, 447–454.

[103] H. U. C. Brass, A. S. Klein, S. Nyholt, T. Classen, J. Pietruszka, *Adv. Synth. Catal.* **2019**.
[104] Z. You, X. Liu, S. Zhang, Y. Wang, *Biochem. Eng. J.* **2018**, *134*, 1–11.
[105] A. de Luna-Preitschopf, H. Zwickl, S. Nehrer, M. Hengstschläger, M. Mikula, *Int. J. Mol. Sci.* **2017**, *18*.
[106] E. I. Graziani, F. V. Ritacco, M. Y. Summers, T. M. Zabriskie, K. Yu, V. S. Bernan, M. Greenstein, G. T. Carter, *Org. Lett.* **2003**, *5*, 2385–2388.
[107] P. A. S. Lowden, G. A. Böhm, S. Metcalfe, J. Staunton, P. F. Leadlay, *Chembiochem* **2004**, *5*, 535–538.
[108] F. V. Ritacco, E. I. Graziani, M. Y. Summers, T. M. Zabriskie, K. Yu, V. S. Bernan, G. T. Carter, M. Greenstein, *Appl. Environ. Microbiol.* **2005**, *71*, 1971–1976.
[109] L. E. Khaw, G. A. Böhm, S. Metcalfe, J. Staunton, P. F. Leadlay, *J. Bacteriol.* **1998**, *180*, 809–814.
[110] M. A. Gregory, H. Petkovic, R. E. Lill, S. J. Moss, B. Wilkinson, S. Gaisser, P. F. Leadlay, R. M. Sheridan, *Angew. Chem. Int. Ed. Engl.* **2005**, *44*, 4757–4760.
[111] H. Nishida, T. Sakakibara, F. Aoki, T. Saito, K. Ichikawa, T. Inagaki, Y. Kojima, Y. Yamauchi, L. H. Huang, M. A. Guadliana, *J. Antibiot.* **1995**, *48*, 657–666.
[112] H. Kanda, K. Yamawaki, *Clin. Exp. Nephrol.* **2020**, *24*, 857–864.
[113] G. Francis, Z. Kerem, H. P. S. Makkar, K. Becker, *Br. J. Nutr.* **2002**, *88*, 587–605.
[114] C. Li, C. Fan, J. Zhao, M. Di, C. Sui, L. Han, L. Hu, *Inflammation* **2020**.
[115] X. Yan, Y. Fan, W. Wei, P. Wang, Q. Liu, Y. Wei, L. Zhang, G. Zhao, J. Yue, Z. Zhou, *Cell Res.* **2014**, *24*, 770–773.
[116] T. Moses, J. Pollier, L. Almagro, D. Buyst, M. van Montagu, M. A. Pedreño, J. C. Martins, J. M. Thevelein, A. Goossens, *Proc. Natl. Acad. Sci. USA* **2014**, *111*, 1634–1639.
[117] X.-D. Yang, Y.-Y. Yang, D.-S. Ouyang, G.-P. Yang, *Fitoterapia* **2015**, *100*, 208–220.
[118] S.-E. Park, C.-S. Na, S.-A. Yoo, S.-H. Seo, H.-S. Son, *J. Ginseng Res.* **2017**, *41*, 36–42.
[119] L. Feng, C. Xu, Z. Li, J. Li, Y. Dai, H. Han, S. Yu, S. Liu, *Prep. Biochem. Biotechnol.* **2016**, *46*, 336–341.
[120] Y. Jin, S. Y. Jung, Y.-J. Kim, D.-Y. Lee, V. C. Aceituno, C. Wang, D.-C. Yang, *Antonie van Leeuwenhoek* **2016**, *109*, 179–185.
[121] L.-H. Quan, J.-W. Min, Y. Jin, C. Wang, Y.-J. Kim, D.-C. Yang, *J. Agr. Food Chem.* **2012**, *60*, 3776–3781.
[122] S.-H. Kim, J.-W. Min, L.-H. Quan, S. Lee, D.-U. Yang, D.-C. Yang, *J. Ginseng Res.* **2012**, *36*, 291–297.
[123] L.-H. Quan, Y.-J. Kim, G. H. Li, K.-T. Choi, D.-C. Yang, *World J. Microbiol. Biotechnol.* **2013**, *29*, 1001–1007.
[124] S. Annett, G. Moore, T. Robson, *Pharmacol. Ther.* **2020**, *215*, 107623.
[125] S. Mo et al., *J. Am. Chem. Soc.* **2011**, *133*, 976–985.
[126] Y. H. Ban, J. H. Lee, G. R. Gu, B. Lee, S. Mo, H. J. Kwon, Y. J. Yoon, *Mol. Biosyst.* **2013**, *9*, 944–947.
[127] D. H. Kim, J. H. Ryu, K. S. Lee, B. M. Lee, M. O. Lee, S.-K. Lim, P. J. Maeng, *Appl. Microbiol. Biotechnol.* **2013**, *97*, 5881–5892.
[128] S. J. Moss et al., *Med. Chem. Commun.* **2013**, *4*, 324–331.
[129] A. Lechner, M. C. Wilson, Y. H. Ban, J.-Y. Hwang, Y. J. Yoon, B. S. Moore, *ACS Synth. Biol.* **2013**, *2*, 379–383.
[130] T. S. Chen, B. H. Arison, L. S. Wicker, E. S. Inamine, R. L. Monaghan, *J. Antibiot.* **1992**, *45*, 118–123.
[131] B. R. Petuch, B. Arison, A. Hsu, R. Monaghan, F. J. Dumont, T. S. Chen, *J. Ind. Microbiol. Biot.* **1994**, *13*, 131–135.
